# SARS Transmission and Commercial Aircraft

**DOI:** 10.3201/eid1008.040093

**Published:** 2004-08

**Authors:** J. Gabrielle Breugelmans, Phillip Zucs, Klaudia Porten, Susanne Broll, Matthias Niedrig, Andrea Ammon, Gérard Krause

**Affiliations:** * Robert Koch-Institut, Berlin, Germany

**Keywords:** letter, severe acute respiratory syndrome, transmission, aircraft, cohort studies

**To the Editor:** Severe acute respiratory syndrome (SARS) is an emerging transmissible disease first reported in Asia in February 2003. The disease is characterized by acute onset of fever with nonproductive cough, myalgia, shortness of breath, or difficulty breathing (1). Approximately 14% of case-patients require mechanical ventilation (1,2). The syndrome is caused by the previously unrecognized SARS-associated coronavirus (SARS-CoV) (3). The primary mode of SARS transmission is through close person-to-person contact. In March 2003, the World Health Organization (WHO) issued two travel advisories to SARS-affected countries. Despite these advisories, probable case-patients traveled by air internationally, thereby spreading the disease globally. The extent of risk posed by probable cases for in-flight transmission of SARS is unclear.

A study was conducted by the Robert Koch Institute in Berlin, Germany, to document SARS transmission during international flights. On April 11, 2003, the Institute was notified that a probable SARS-infected person had flown from Hong Kong to Frankfurt, Germany, on March 30 to 31, 2003, and then traveled extensively in Europe after onset of symptoms. In 5 days, the traveler, a 48-year-old Hong Kong businessman, had flown on seven flights throughout Europe (Table). On March 31, symptoms of SARS, including fever and general malaise developed; whether he had a cough at this time is unclear. He was admitted to a hospital in Hong Kong on April 8, and mechanical ventilation was initiated. He was reported to WHO as a suspected SARS patient on April 9 and diagnosed with SARS on April 10. Polymerase chain reaction analysis conducted on the patient's nasopharyngeal aspirate showed positive results for SARS-CoV on April 14.

**Table:** Flight itinerary of SARS patient^a^

Departure city	Arrival city	Date/time departure	Date/time arrival	Duration (h:min)
Hong Kong	Frankfurt	March 30/23:10	March 31/05:35	12:25
Frankfurt	Barcelona	March 31/09:05	March 31/11:10	2:05
Onset of symptoms after arrival in Barcelona on March 31, 2003					
Barcelona	Frankfurt	April 2/07:05	April 2/09:15	2:10	
Frankfurt	London	April 2/10:15	April 2/11:30	2:15	
London	Munich	April 3/15:25	April 3/18:10	1:45	
Munich	Frankfurt	April 4/14:50	April 4/16:00	1:10	
Frankfurt	Hong Kong	April 4/17:40	April 5/10:35	9:55	

^a^SARS, severe acute respiratory syndrome.

Passenger manifests from the seven flights on which the patient had flown were requested by the local health departments and the Institute. In a previous study, Kenyon et al. indicated that airline passengers seated within two rows of an infectious tuberculosis patient were at greatest risk for infection (4). To determine an association between seating proximity to the SARS patient and transmission of SARS, a study that included all airline passengers seated within four rows (i.e., front, back, and same row) of the index patient (4) was conducted. Passengers >18 years of age who lived in Germany were contacted by the Institute and asked to participate in the study; all participants gave informed consent for inclusion in the study. Passengers in other countries were not included in the study because contact information was not available. Passengers <18 years of age were not included in the study; ethical approval from an Institutional Review Board, which would have delayed the study, would have been necessary. Contact information for study participants was forwarded to local health departments so that public health officials could provide follow-up care. Study participants were interviewed approximately 3 months after their flights because contact information was not available earlier. A standardized questionnaire was developed to collect information on demographics, flight details, countries visited before the flights, use of mask, and symptoms. Furthermore, 5–10 mL of whole blood was drawn and tested for SARS-CoV antibodies by using immunofluorescence assay.

A total of 250 passengers were identified and selected for the study. Contact information was available for 109 passengers; 69 of the 109 were living in Germany. Sixty-two of those 69 passengers were contacted, and 41 passengers agreed to participate in the study. Thirty-six participants completed questionnaires and had blood samples taken. The male-to-female ratio was 3:5, and the median age was 41 years (25–59 years). Contact information was not available for five passengers, which made their inclusion in the study impossible. All serologic samples (N = 36) tested were negative for SARS-CoV immunoglobulin G antibodies, and none of the 36 passengers reported symptoms characteristic of SARS. Ten passengers complained of cough, headache, and muscle aches. One passenger reported a cough, muscle aches, and fever, but symptoms started 10 days after the flight. An analysis of the seating arrangement showed that the study participants were randomly distributed around the index patient.

No SARS transmission was shown among contacted passengers seated in close proximity to the index patient; these results suggest that in-flight transmission of SARS is not common. These results are consistent with other studies that assessed the risk for in-flight transmission of SARS (5,6). The results also suggest that SARS-CoV is not efficiently transmitted, as reflected in its basic reproduction number R_0_ (range 2–4) (7). The SARS-infected patient on the indicated flights was in his first week of illness; infectivity is greatest in the second week (8). Therefore, the likelihood of SARS transmission on the indicated flights was not high. These results are further supported by the fact that all contacts were asymptomatic 13 days after their last contact with the SARS patient. No information was available on healthcare contacts. Although we did not observe any SARS transmission, we cannot rule out the possibility that it may have occurred. We had no contact information on 56% of the passengers on the indicated flights and, therefore, had to exclude them from the investigation. Obtaining complete contact information from the remaining passengers was difficult, which severely impeded the investigation. Similarly, we were unable to contact crew members and had to exclude them. Recent studies have documented SARS transmission to passengers seated more than four rows away from an index patient (5,9); thus, studying the passenger proximity to the patient may not be sufficient. Because of these limitations, our final sample size was small and probably biased. Since we did not observe any evidence to indicate in-flight transmission of SARS, we were unable to assess the importance of seat assignment proximity as a risk factor.

The study shows that the roles of public health authorities and the aviation industry should be to "harmonise the protection of public health without the need to avoid unnecessary disruption of trade and travel" in public health emergencies such as global SARS transmission (10). We recommend strengthening the collaboration between national health authorities and the airline industry. Furthermore, the International Air Transport Association should establish procedures to ensure that complete contact information is available for all passengers and that rapid notification can be accomplished in case of potential exposure to infectious diseases.
